# Septo-temporal distribution and lineage progression of hippocampal neurogenesis in a primate (*Callithrix jacchus*) in comparison to mice

**DOI:** 10.3389/fnana.2015.00085

**Published:** 2015-06-29

**Authors:** Irmgard Amrein, Michael Nosswitz, Lutz Slomianka, R. Maarten van Dijk, Stefanie Engler, Fabienne Klaus, Olivier Raineteau, Kasum Azim

**Affiliations:** ^1^Functional Neuroanatomy, Institute of Anatomy, University of ZürichZürich, Switzerland; ^2^Neuroscience Center Zurich, University of Zürich and ETH ZürichZürich, Switzerland; ^3^Institute of Human Movement Sciences and Sport, Department of Health Sciences and Technology, ETH ZürichZürich, Switzerland; ^4^Inserm U846, Stem Cell and Brain Research Institute, BronFrance; ^5^Université de Lyon, BronFrance

**Keywords:** *Callithrix jacchus*, marmoset, neurogenesis, transcription factors, hippocampus, septo-temporal, comparative

## Abstract

Adult born neurons in the hippocampus show species-specific differences in their numbers, the pace of their maturation and their spatial distribution. Here, we present quantitative data on adult hippocampal neurogenesis in a New World primate, the common marmoset (*Callithrix jacchus*) that demonstrate parts of the lineage progression and age-related changes. Proliferation was largely (∼70%) restricted to stem cells or early progenitor cells, whilst the remainder of the cycling pool could be assigned almost exclusively to Tbr2+ intermediate precursor cells in both neonate and adult animals (20–122 months). Proliferating DCX+ neuroblasts were virtually absent in adults, although rare MCM2+/DCX+ co-expression revealed a small, persisting proliferative potential. Co-expression of DCX with calretinin was very limited in marmosets, suggesting that these markers label distinct maturational stages. In adult marmosets, numbers of MCM2+, Ki67+, and significantly Tbr2+, DCX+, and CR+ cells declined with age. The distributions of granule cells, proliferating cells and DCX+ young neurons along the hippocampal longitudinal axis were equal in marmosets and mice. In both species, a gradient along the hippocampal septo-temporal axis was apparent for DCX+ and resident granule cells. Both cell numbers are higher septally than temporally, whilst proliferating cells were evenly distributed along this axis. Relative to resident granule cells, however, the ratio of proliferating cells and DCX+ neurons remained constant in the septal, middle, and temporal hippocampus. In marmosets, the extended phase of the maturation of young neurons that characterizes primate hippocampal neurogenesis was due to the extension in a large CR+/DCX- cell population. This clear dissociation between DCX+ and CR+ young neurons has not been reported for other species and may therefore represent a key primate-specific feature of adult hippocampal neurogenesis.

## Introduction

The common marmoset (*Callithrix jacchus*) has become a widely used simian primate species in neuroscience research due to its small size and easy handling, its primarily lissencephalic yet relative large brain and high birth rate (usually non-identical twins, occasionally triplets Ross, 1991). Research areas range from cognitive studies (Burkart et al., 2009, 2014) to systems neuroscience (e.g., Solomon and Rosa, 2014) and neurological disease models (for review see Okano et al., 2012). The generation of transgenic marmoset models (Sasaki et al., 2009) and efforts to create detailed marmoset brain maps using MRI, fMRI, and DTI (Okano and Mitra, 2014) further increases the usefulness of this primate.

Adult neurogenesis in primates shows features not observed in other genera. Differences are numerous for the neurogenic niche of the subventricular zone (SVZ), rostral migratory stream (RMS) and olfactory bulb (Bonfanti and Peretto, 2011). For example, the SVZ micro-domain heterogeneity observed in rodents is apparent only in early postnatal life in marmosets (Azim et al., 2013), and migration of neuroblasts to the RMS and olfactory bulb has a ventral origin that shows a very rapid postnatal decline to near or complete disappearance as in other primates and humans (Sanai et al., 2011; Bergmann et al., 2012; Azim et al., 2013). In addition, dividing precursor cells have been shown *in vivo* and *in vitro* in the early postnatal marmoset visual cortex (Homman-Ludiye et al., 2012). The neurogenic niche in the hippocampus also shows primate-specific traits. While reports in rhesus and cynomolgus monkeys suggest that newborn cells pass through a sequence of developmental stages similar to that in rodents, the maturation of young neurons is markedly slower in primates (Ngwenya et al., 2006; Taffe et al., 2010; Aizawa et al., 2011; Kohler et al., 2011). Furthermore, the rate of hippocampal neurogenesis is considered to be lower in primates than rodents (Kornack and Rakic, 1999; Jabès et al., 2010; Kohler et al., 2011), although differences may stem from methodological differences or relate to the considerably longer life span of primates (Amrein et al., 2011). Defining the functional significance of hippocampal neurogenesis in primates is complicated by differences in the relative positioning of the hippocampus in rodents and primates. While the hippocampus in primates and humans is a relatively straight structure in the temporal lobe, it has a bent structure in rodents arching first laterally and then ventrally from the septum to its junction with the amygdala at the temporal pole. In addition, anatomical gradients that are superimposed to segregated gene expression and intrinsic connection profiles, both in rodents and primates, have been reported along the long axis of the hippocampus (reviewed by Fanselow and Dong, 2010; Strange et al., 2014). In the human hippocampus, additional functional partitions between anterior (temporal) and posterior (septal) hippocampal regions have been proposed (Poppenk et al., 2013).

In this study, design-based quantitative stereological methods were used to investigate neurogenesis in the hippocampal formation of the common marmoset. We assessed the numbers of resident granule cells, Ki67+ proliferating cells (Starborg et al., 1996) and DCX+ young neurons (Gleeson et al., 1999) along the septo-temporal axis. To compare distributions in a primate and a rodent hippocampus, Ki67+ cells, DCX+ young neurons and granule cells were also investigated in hippocampi of C57BL/6 mice that were straightened to approximate the shape of the hippocampus in the primate brain. This methodological approach overcomes the topographical difficulties of *in situ* definitions (Tanti and Belzung, 2013) by allowing direct comparisons of septo-temporal cell distributions in the marmoset and mouse dentate gyrus. Furthermore, we quantitatively characterized aspects of lineage progression in marmosets (neonates and up to an age of 122 months) by estimating the numbers of proliferating, Ki67+ cells co-expressing DCX, MCM2 (minichromosome maintenance complex component 2; a protein essential for the pre-replication complex, Tye, 1999) or Tbr2 (a T-domain transcription factor expressed by intermediate precursor cells, Englund et al., 2005) and by estimating the numbers of maturing, DCX+ granule cells co-expressing MCM2 or calretinin, which is transiently expressed in immature neurons (Brandt et al., 2003). Findings in marmosets are compared with rodent data to provide a quantitative framework for similarities and divergent traits.

## Materials and Methods

### Animals

Seven male and four female common marmosets, aged between postnatal day 0 (neonates) and 10 years were investigated. Adult animals had a mean bodyweight of 400 g and mean brain weight of 8 g, whereas neonates had mean bodyweight of 31 g and brain weight of 3.4 g. Animals were euthanized with 10 mg/kg bodyweight ketamine and 0.5 mg/kg bodyweight xylazine. Postmortem tissue harvesting was performed in agreement with Canton of Zurich veterinary office guidelines. Upon cardiac arrest, the chest was opened and the animals were transcardially perfused with heparinized phosphate buffered saline (PBS, pH 7.4), followed by 0.6% sodium sulfide in phosphate buffer and, finally, cold 4% paraformaldehyde (PFA) solution in PBS containing 15% picric acid (PFA-PA). Brains were removed, weighted, separated into hemispheres and post-fixed for 24 h in PFA-PA. Right hemispheres were conserved in fresh PFA-PA for HEMA embedding (see below). Left hemispheres were transferred into 30% sucrose solution and processed for brightfield and fluorescence immunohistochemistry.

Ten male C57BL/6 mice (OlaHsd, Harlan, NL), aged 14 weeks, were sacrificed by an overdose of pentobarbital (50 mg/kg) and perfused transcardially with cold PBS followed by cold 1% PFA-PA. Brains were removed rapidly and the hippocampi dissected. Isolated left and right hippocampi were gently straightened and fixed with 4% PFA-PA in grooves (25 mm × 3 mm × 4 mm) carved into PVC blocks. Hippocampi were post-fixed in this straightened position for 3 h, during which PFA-PA was exchanged every hour.

### Histology and Immunohistochemistry in Marmosets

Right hemispheres were separated into a frontal, middle and occipital block, with the middle block containing the entire hippocampal formation. Blocks were dehydrated and embedded in HEMA (2-hydroxyethyl methacrylate; Technovit 7100, Heraeus Kulzer GmbH, Wehrheim/Ts, Germany) following the manufacturer’s instruction, but with extended infiltration times. Series of every sixth 20 μm thick coronal section were mounted and dried at 60°C for at least 1 h. One series was Giemsa-stained (Giemsa stock solution 1.09204.0500, Merck, Darmstadt, Germany) following the protocol of Iñiguez et al. (1985). Another series (four animals) was Timm-stained (Danscher and Zimmer, 1978).

Left hemispheres were blocked similar to the right hemispheres for immunohistochemistry. From the middle, hippocampus-containing frozen block, 20 series of 40 μm thick coronal sections were collected and stored in cryoprotectant at -20°C until processing. One series was used as reference series, for which sections were collected in order into well plates, mounted and Giemsa stained as described above. Immunohistochemically sections, which were stained free-floating, were mounted in correct order by referring to the reference series.

Details for primary antibodies are listed in **Table 1**. For brightfield immunohistochemistry, free-floating sections were washed in TBS-T [Tris-buffered saline (TBS) pH 7.4 with 0.05% Triton] prior to primary antibody incubation [Ki67, DCX, and calretinin (CR) for details see **Table 1**], afterward with TBS alone. Endogenous peroxidase was blocked with 0.6% H_2_O_2_ in TBS-T for 15 min. The diluent for pre- and incubation of primary antibodies contained 2% normal serum, 0.2% Triton, and 0.1% bovine serum albumin in TBS-T. Afterward, sections were incubated with secondary antibodies (all Vector, 1:300) followed by ABC solution (Vectastain Elite Kits, Vector Laboratories, Burlingame, CA, USA) and visualized using diaminobenzidine (Sigmafast^TM^, D4418-50SET).

**Table 1:** Antibodies, dilutions and antigen-retrieval.

Antibody	Source	Antigen	Dilution	Antigen retrieval
**Marmoset**				
Polyclonal rabbit anti Ki67 IgG	NCL-Ki67-p, Novocastra	Prokaryotic recombinant fusion protein corresponding to a 1086 dp Ki67 motif-containing cDNA fragment	1:2500	Heat treatment for 40 min at 90°C in 1:10 citrate buffer pH6.0 (DAKO REAL Target Retrieval Solution)
Mouse anti-Ki67	BD Pharm	Immunodominant epitope of human Ki67 protein	1:300	See above
Polyclonal goat anti-doublecortin (DCX) IgG	sc-8066, Santa Cruz Biotechnology	Epitope mapping at the C-terminus of human doublecortin	1:300	Microwave treatment at 600 W for 1.5 min in 1:10 citrate buffer pH6.0 (DAKO REAL Target Retrieval Solution)
Polyclonal rabbit anti-Tbr2	AB23345, Abcam	KLH-conjugated linear peptide corresponding to mouse Tbr2	1:1500	See Ki67
MCM2	MCM2 (D7611)xP Rabbit mAB, Cell signaling	Synthetic peptide corresponding to amino-terminal residues of human MCM2	1:50	See Ki67
Ascl (Mash1)	AB 38557, abcam	Synthetic peptide at N-terminus of first 100 amino-acids of human achaete-scute homolog 1 conjugated to KLH	1:50 – 1:500	See Ki67
Monoclonal mouse anti-calretinin IgG	MAB 1568, Millipore	Recombinant rat calretinin	1:2000	See DCX
**C57BL/6 mouse**				
Mouse anti-Ki67	BD Pharm	Immunodominant epitope of human Ki67 protein	1:300	See Ki67 marmoset
Polyclonal goat anti-doublecortin (DCX) IgG	sc-8066, Santa Cruz Biotechnology	Epitope mapping at the C-terminus of human doublecortin	1:250	See DCX marmoset


For fluorescence immunohistochemistry, sections were washed several times with PBS, followed by a 30 min rinse in PBS containing 0.5% Triton (PBS-T). Protocol details are listed in **Table 1**. The following combinations of primary antibodies were used: Ki67/Ascl1, Ki67/Tbr2, Ki67/DCX, Ki67/MCM2, DCX/CR, and DCX/MCM2. Pre-incubation for primary antibodies was made in a diluent of 10% normal horse serum in PBS-T, whereas the incubation solution contained 5% normal horse serum only. Cocktails containing two primary or two secondary antibodies (all Alexa Fluors, 1:400, Invitrogen, in 5% normal horse serum in PBS-T) raised in different species were applied. Nuclear counterstaining was performed with 4′,6-diamidino-2-phenylindole (DAPI, Invitrogen) in PBS. Mounted sections were embedded with ProLong Gold (Life Technologies).

### Matrix-Embedding of Straightened Mouse Hippocampi and Processing

For immunohistochemistry, straightened left hippocampi of C57BL/6 mice were embedded in a gelatine-albumin protein matrix following the protocol of Smiley and Bleiwas (2012). In brief, straightened, fixed hippocampi were cryoprotected by immersion in glycerol. A base layer of protein matrix composed of gelatine-egg-albumin with the cross-linking reagents glutaraldehyde (25% EM grade) and lysine was prepared in molds (25 mm × 20 mm × 14 mm). Hippocampi were positioned in parallel on the base layer (5–6 hippocampi per mold) and gently pushed below the surface. After 10 min, the mold was filled with freshly prepared protein matrix. Matrix blocks containing the embedded hippocampi were then cryoprotected by immersion into 20% glycerol in PBS. Frozen blocks were cut perpendicular to the longitudinal axis of the hippocampi at 40 μm. Series of every 10th section were collected and stored in cryoprotectant until further processing. A reference series was mounted immediately in the correct anatomical order and Giemsa-stained as described for the marmoset tissue. Immunhistochemistry for Ki67 and DCX followed the protocol described for the marmoset tissue (see above and **Table 1**).

For granule cell counts, straightened right hippocampi were dehydrated and embedded in HEMA (see above). Blocks containing 5–6 hippocampi were cut perpendicular to the hippocampal longitudinal axis at 20 μm. Every third section was collected, mounted and Giemsa-stained.

### Quantitative Stereological Procedures

In marmosets, immunopositive cells in the adult animals were counted exhaustively using area and thickness sampling fractions of 1, but omitting cells in the top focal plane. DAB stained Ki67+ and DCX+ cells were each evaluated in two equidistant series of sections, i.e., in every 10th section. Immunofluorescent cells were evaluated in one series, i.e., in every 20th section. Cell counts were multiplied either by 10 or 20 to estimate total cell numbers. Double-positive cells were verified by acquiring image stacks using structured illumination (ApoTome.1, Zeiss, Germany). Numbers of immunopositive cells in the neonate animals were estimated using the optical Fractionator (West et al., 1991) with StereoInvestigator 10 software (MBF Bioscience, Williston, VT, USA). A counting frame size of 35 × 35 μm and x- and y-step sizes of 180 μm were used.

Estimates of total granule cell number were obtained from HEMA-embedded sections using the optical Fractionator. Every 18th section of neonates, and every 24th section of adults was analyzed. Other sampling parameters were the same for all animals. We used disector samples of 15 × 15 × 10 μm and x- and y-step sizes of 120 μm. As described before in foxes (Amrein and Slomianka, 2010), different granule cell types were observed and assessed separately: large-sized granule cells with usually one distinct and large nucleolus and a large cytoplasm, and small-sized granule cells with less distinct small or multiple small nucleoli and very narrow rims of cytoplasm. Apoptotic cells were identified by their morphology (condensed chromatin, C-shaped nucleus to fragmented dense nuclear bodies surrounded by a halo of pale cytoplasm, Sloviter et al., 1993; Amrein et al., 2004) and counted exhaustively in every sixth HEMA-embedded section because of their low numbers. Volumes of granule cells, CR+ and DCX+ cells were assessed using the Nucleator (Gundersen, 1988) with four test lines with StereoInvestigator 10 software. Coefficients of error (CE) for *m* = 0 were calculated (Gundersen et al., 1999; Slomianka and West, 2005) to assess estimate precisions (**Table 2**).

**Table 2:** Estimated cell numbers in neurogenesis.

Animal	Sex	Age	MCM2+ ^∗^	Ki67+ ^∗^	Tbr2+	DCX+ ^∗^	CR+	MCM2+/Ki67+	Tbr2+/Ki67+	DCX+/Ki67+	MCM2+/DCX+	CR+/DCX+	Apoptotic cell
**Marmoset**					
C 05 s	F	0	257080	94740	142720	NA	NA	97330	40960	NA	NA	NA	NA
C 06 s	F	0	224290	62050	76700	NA	NA	79880	20630	NA	NA	NA	NA
C 09 s	M	20	5050	5700	5360	21220	8300	4660	2240	0	120	80	156
C 11 s	F	20	8140	9830	9460	35400	8000	6500	3380	0	400	200	168
C 01	M	31.25	1120	750	1060	7250	11180	700	80	0	20	100	150
C 02 s	M	32	1710	1300	2740	12780	11380	1040	560	20	60	40	186
C 03 s	M	32	1090	920	1180	8250	12780	680	200	0	60	20	210
C 07	M	46.5	1190	760	840	2950	8360	600	180	0	100	0	60
C 04	M	56.25	600	220	580	5730	7920	220	40	0	80	0	120
C 10	F	63.5	1660	950	580	3780	5700	1240	280	0	180	40	60
C 08	M	122.5	730	650	480	1830	5540	560	200	0	40	0	24
**Mouse**													
C57BL/6	M	3.5	NA	5820	NA	14440	NA	NA	NA	NA	NA	NA	NA


*Total estimated numbers of immunopositive cells in marmosets and mice. Marmosets are ordered according to age (in months), siblings are marked with ‘s’. Estimated total numbers are given for one hemisphere (unilateral) and rounded to the next 10. ^∗^Cell estimates are given as mean numbers of independently assessed quantification from different immunomarker combinations for Ki67 (four), DCX (three) and MCM2 (two). Calculated coefficient of error (CE) in marmoset was for Ki67: CE = 0.07 (0.03–0.12); DCX: CE = 0.06 (0.04–0.08); Apoptotic cells: 0.14 (0.08–0.34). In mice coefficient of error (CE) was for Ki67: CE = 0.06; and DCX: CE = 0.08. Data of mice represent means of 10 animals. NA, not assessed.*

In C57BL/6 mice, DCX+ young neuron numbers were estimated in every 10th section with the optical Fractionator using a counting frame size of 35 × 35 μm and step sizes of 75 μm. Ki67+ cells were counted exhaustively in every 10th section. Again, stained cells in the top focal plane were not considered. Total cell number estimates were calculated by multiplying cell counts by 10.

Granule cells were estimated in every 12th HEMA embedded section, using the optical fractionator with disector samples sized 10 × 10 × 10 μm and step sizes of 110 μm.

### Morphing the Hippocampus

The numbers of granule cells, Ki67+ and DCX+ cells in the marmoset and mouse hippocampus were collected in the correct anatomical order. For visualization of the septo-temporal cell distribution, we processed the data as described before (Slomianka and West, 1987). In short, the length along the septo-temporal axis was standardized for all animals by selecting a number of virtual sections as a standard length. We choose 14 virtual sections, which corresponded to the integer closest to the mean number of sections analyzed to minimize data dilution or loss. The count obtained in each real section was divided into a number of sub-bins that corresponded to the desired number of virtual sections. Numbers for virtual sections were generated by successively pooling a number of these sub-bins. The number of pooled sub-bins corresponded to the number of the real sections available for an animal. E.g., three sections with counts of 8, 12, and 6 shall be re-binned into four virtual sections: counts are divided into the sub-bins 2, 2, 2, 2, 3, 3, 3, 3, 1.5, 1.5, 1.5, and 1.5. Three sub-bins are sequentially aggregated into four virtual sections with counts of 6, 8, 7.5, and 4.5.

### Statistics

In marmosets, the numbers of proliferating, Ki67+ cells were estimated in four different series, doublecortin (DCX) in three different series, and MCM2 in two different series. When multiple estimates of one cell population were available, the means of the estimates were used for further statistical analysis. Statistical analysis of the age-dependent regulation of cell numbers in marmosets was performed with SPSS Version 20 using the curve estimation regression model with cell numbers as dependent and age in months as independent variable. Both linear and exponential models were tested and best fit data are presented. General linear models (GLMs) with log-transformed neurogenesis-related cell counts as a ratio of total granule cells (normalized values) as dependent variable, order (primate and rodent) as fixed factors, and age as covariate was used to test primate-rodent differences in neurogenesis. Statistical analyses of the morphed cell data in marmosets and mice along the septo-temporal axis were conducted with R version 3.0.3 (Ihaka and Gentleman, 1996; R Core Team, 2014). A repeated one-way ANOVA was used to test for cell number differences along the septo-temporal axis. *Post hoc* comparisons were made using the Tukey test. For this analysis, we excluded the first and last virtual section because of the large variability in these virtual sections related to the uniform random systematic selection of real sections. The remaining 12 sections were divided into three parts: temporal (sections 2–5), middle (sections 6–9), and septal (sections 10–13). Graphical presentations were made with the R package ggplot2 (Wickham, 2009).

## Results

### Comparison of Cell Distribution along the Hippocampal Long Axis in Adult Marmosets and Mice

**FIGURE 1:**
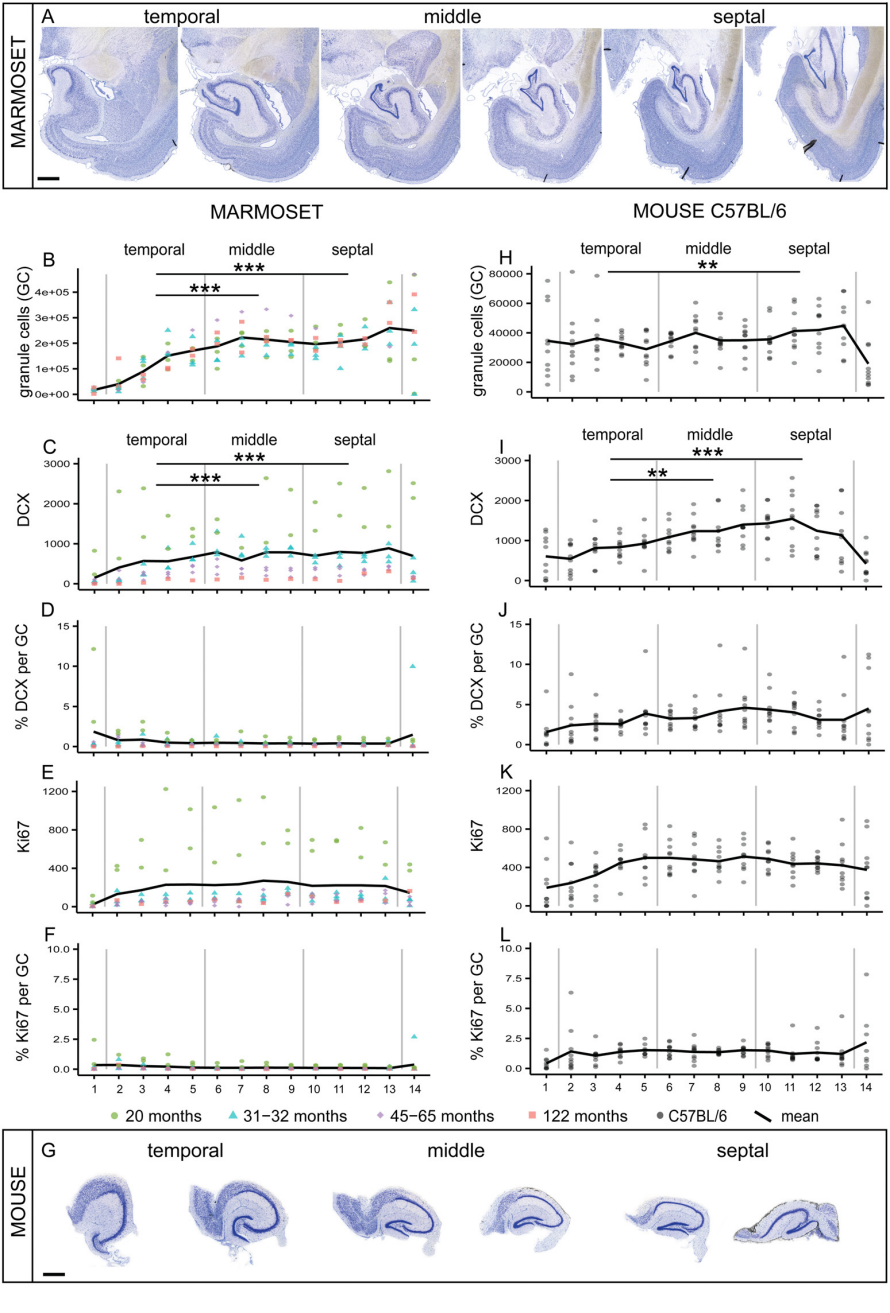
**Comparison of septo-temporal gradients in marmoset and mouse.(A)** Giemsa-stained coronal sections from the temporal to the septal pool of a marmoset hippocampus (distance between sections 960 μm). Significant gradients along the septo-temporal axis are found in morphed numbers of granule cells (GC, **B**) and DCX **(C)**. Proliferating cells (Ki67, **E**) and normalized neurogenesis-related cell numbers expressed as a percentage of local granule cells (% DCX per GC, **D**; and % Ki67 per GC, **F**) are evenly distributed along the longitudinal axis. **(G)** Coronal sections of the matrix-embedded, straightened hippocampus of a C57BL/6 mouse (distance between sections 120 μm). Similar to the marmoset hippocampus, a significant septo-temporal gradient is apparent for granule cells **(H)** and DCX+ cells **(I)**, but not for the other cell numbers **(J–L)**. All stereologically assessed cell numbers are morphed to 14 virtual sections, section 1 and 14 were excluded from the statistical analysis. Numbers for marmosets are color-coded for individual ages, all C57BL/6 (*N* = 10) are 14 weeks of age. For exact *p*-values see result section. Scale bar **(A)**: 1 mm; **(G)**: 500 μm.

Granule cell numbers in the adult marmoset hippocampus (**Figure 1A**) increased significantly from the temporal to the septal end of the hippocampus [*F*(1,8) = 38.96, *p* < 0.001, **Figure 1B**]. *Post hoc* comparison indicated that the temporal part harbors less granule cells than the middle (*p* < 0.001) and septal (*p* < 0.001) third, whereas the middle and septal part did not differ from each other. A septo-temporal gradient was also apparent for the DCX+ young neurons [*F*(1,8) = 16.25, *p* = 0.004, **Figure 1C**]. *Post hoc* analysis again showed that DCX+ cell numbers were lower in the temporal third than in the middle (*p* < 0.001) and septal (*p* < 0.001) third. In contrast, proliferating Ki67+ cells were evenly distributed along the septo-temporal axis [*F*(1,8) = 1.69, *p* = 0.23, **Figure 1E**]. Presented as a percentage of local granule cells, there was no significant septo-temporal gradient for DCX+ [*F*_RatioDCX/GC_(1,8) = 4.51, *p* = 0.066, **Figure 1D**] or proliferating cells [*F*_RatioKi67/GC_(1,8) = 3.14, *p* = 0.11, **Figure 1F**].

In the extended mouse hippocampus (**Figure 1G**), the septo-temporal distribution of cell numbers followed the same patterns as in the marmosets, i.e., cell numbers were lower temporally than septally. Gradients were found for the numbers of granule cells [*F*(1,9) = 6.4, *p* = 0.032, **Figure 1H**] and for DCX+ young neurons [*F*(1,9) = 9.53, *p* = 0.013, **Figure 1I**], but not for Ki67+ cells [*F*(1,9) = 2.56, *p* = 0.144, **Figure 1K**] or the normalized cell numbers [*F*_RatioDCX/GC_(1,9) = 2.5, *p* = 0.15, **Figure 1J**; *F*_RatioKi67/GC_(1,9) = 0, *p* = 0.98, **Figure 1L**]. *Post hoc* comparisons of the distribution of granule cells in mice indicated that the temporal third was significantly different from the septal third (*p* = 0.007), whereas DCX+ cell numbers in the temporal third differed significantly from the middle (*p* = 0.003) and the septal (*p* < 0.001) third.

### Age-Related Changes in Dentate Gyrus Cell Populations in Marmosets

#### MCM2+ Cells with Replication Potential

**FIGURE 2:**
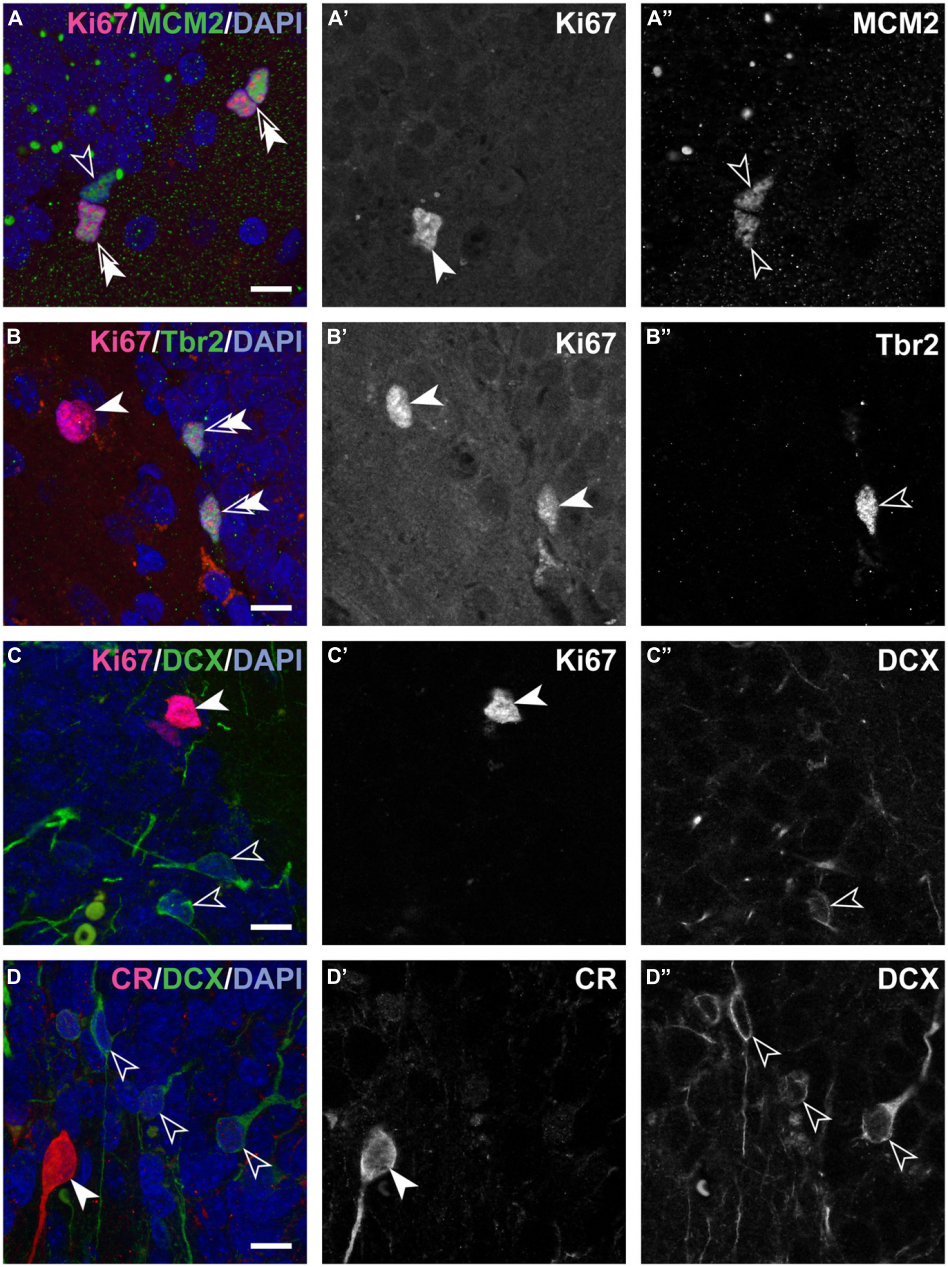
**Composite and single channel images of representative immunostained cells in the adult marmosets.** Co-localization of various markers are visualized in composite pictures of ApoTome stacks for double immunostainings showing Ki67 and MCM2 **(A)**, Ki67 and Tbr2 **(B)**, Ki67 and DCX **(C)** and last DCX and CR **(D)**. Note that colored composite pictures are maximum intensity projections of at least 30 optical planes with distance of 0.2 μm between planes. Instead, monochromatic (grayscale) **(A’,A”,B’,B”,C’,C”**, and **D’,D”)** are images from a single focal plane, separated for the two excitation channels used. Thus, some positive cells in the composite picture are above or below the section plane in the monochromatic pictures. Note: spectral bleed-through of the signals was not observed. Double arrows in the composite images indicate cells positive for both markers. Scale bar: 10 μm for all images.

The number of MCM2+ cells (**Table 2**; **Figure 2A”**) declined when neonates are included in the analysis [*b* = -0.69, *t*(10) = -2.9, *p* = 0.018], but appeared relatively stable throughout adulthood [*b* = -0.6, *t*(8) = -1.97, *p* = 0.09].

#### Ki67+ Proliferating Cells

In the hippocampus of neonate marmosets, cell division was prominent, and these cells accounted for 6.8% of all granule cells. In the adult marmosets, this value declined from 0.23% in 20 months old to 0.02% in the oldest animal (**Table 2**; **Figures 2A’,B’,C’**). The age-related decline of proliferating cells was significant when tested across all ages [*b* = -0.7, *t*(10) = -29, *p* = 0.017], but appeared stable when only adult animals were included [*b* = -0.52, *t*(8) = -1.6, *p* = 0.15]. Similarly, the number of actively proliferating MCM2+ cells (MCM2+/Ki67+ cells, **Figure 2A**) declined across all animals [*b* = -0.7, *t*(10) = -2.8, *p* = 0.021], but not if only adult animals were considered [*b* = -0.5, *t*(7) = -1.5, *p* = 0.18].

#### Tbr2+ Intermediate Precursor Cells (IPCs)

The number of Tbr2+ (**Table 2**; **Figure 2B”**) cells showed a steady age-related decline, both when neonates were included [*b* = -0.8, *t*(9) = -3.6, *p* = 0.006] and when only adults were considered [*b* = -0.71, *t*(8) = -2.7, *p* = 0.031].

#### Immature Neurons

Numbers of DCX+ young neurons (**Table 2**; **Figures 2C”,D”**) could not be estimated in neonates as the intense labeling hindered a reliable characterization of single negative cells. In 20 months-old marmosets, DCX+ young neurons accounted for 1.35% of the resident granule cells. Their relative numbers diminished to 0.06% in the oldest animal in this sample, corresponding to a ∼22-fold decline that also resulted in a significant age-related decline [*b* = -0.82, *t*(8) = -3.75, *p* = 0.007]. The number of CR+ (**Figure 2D’**; **Table 2**) cells were assessed in adults only and declined with age [*b* = -0.67, *t*(8) = -2.6, *p* = 0.037].

#### Apoptosis

Numbers of apoptotic cells showed a decline with age in adult animals [*b* = -0.903, *t*(8) = -5.6, *p* = 0.001]. Absolute numbers were, however, very low, resulting in rather high CE values (see **Table 2**). Due to the high variance introduced by the quantitative procedure, further statistical comparisons were not performed.

#### Granule Cells

**FIGURE 3:**
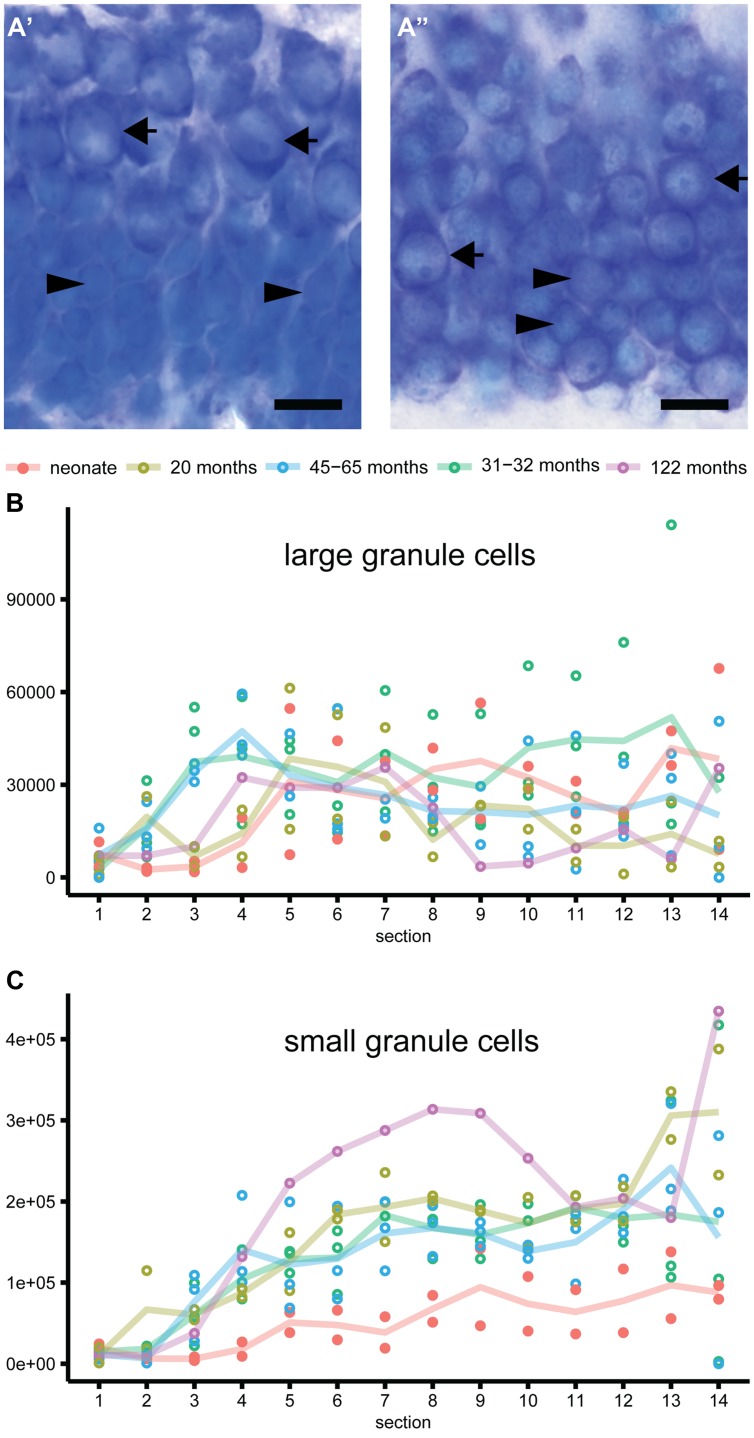
**Heterogeneity of granule cells. (A)** Representative images of the Giemsa stained granule cell layer in neonate **(A’)** and adult **(A”)** marmosets. Distinct morphology of large granule cells with one distinct nucleolus in an otherwise heterochromatin-scarce nucleus and clear cytoplasmic rim (arrow) compared to small granule cells with less distinct small or multiple small nucleoli and narrow rim of cytoplasm (arrowhead) can be observed in neonate and adult marmosets. Whereas the large granule cells are restricted to the outer granule cell layer in neonates, these cells are intermingled with small granule cells in adults. **(B)** Large granule cells are evenly distributed along the septo-temporal axis and do not differ in absolute numbers between neonate and adult marmosets. **(C)** Small-sized granule cells accumulate while aging, and show a strong septo-temporal gradient in the marmoset dentate gyrus.

Newborn marmosets harbored less than half of the granule cells that adults do (**Table 3**; **Figure 3**). Across all animals there were age-dependent increases [*b* = 0.69, *t*(10) = 2.9, *p* = 0.018]. This was also evident for the separately assessed small granule cells [*b* = 0.71, *t*(10) = 3.0, *p* = 0.015, see **Figure 3C**], but not for the large granule cells [*b* = -0.13, *t*(10) = -0.4, *p* = 0.7, **Figure 3B**]. In the adult animals, granule cell numbers remained stable [total granule cells: *b* = 0.51, *t*(8) = 1.56, *p* = 0.163; small granule cells: *b* = 0.55, *t*(8) = 1.73, *p* = 0.13; large granule cells: *b* = -0.19, *t*(8) = -0.51 *p* = 0.62].

**Table 3:** Numbers and volumes of dentate gyrus granule cells.

Animal	Sex	Age	Total granule cell	CE granule cell	Large granule cell	CE Large granule cell	Mean volume (μm^3^) large granule cell	Small granule cell	CE small granule cell	Mean volume (μm^3^) large granule cell
**Marmoset**					
C 05 s	F	0	1026000	0.10	506000	0.12	265 (*N* = 248)	620000	0.10	124 (*N* = 388)
C 06 s	F	0	1156000	0.09	278000	0.16	344 (*N* = 143)	878000	0.08	166 (*N* = 452)
C 09 s	M	20	2467000	0.07	343000	0.13	298 (*N* = 25)	2124000	0.07	204 (*N* = 30)
C 11 s	F	20	2624000	0.08	156000	0.19	NA	2468000	0.08	NA
C 01	M	31.25	2102000	0.06	388000	0.11	315 (*N* = 134)	1714000	0.06	195 (*N* = 590)
C 02 s	M	32	2193000	0.13	313000	0.15	NA	1880000	0.14	NA
C 03 s	M	32	2727000	0.08	719000	0.09	382 (*N* = 238)	2007000	0.08	251 (*N* = 664)
C 07	M	46.5	1891000	0.05	344000	0.10	410 (*N* = 111)	1547000	0.09	225 (*N* = 499)
C 04	M	56.25	2291000	0.09	284000	0.12	350 (*N* = 91)	2007000	0.09	207 (*N* = 643)
C 10	F	63.5	2419000	0.07	417000	0.11	NA	2003000	0.08	NA
C 08	M	122.5	3092000	0.08	244000	0.16	217 (*N* = 139)	2848000	0.08	133 (*N* = 507)
**Mouse**					
C57BL/6	M	3.5	492000	0.08						


*Estimates of total granule cell numbers in marmosets and mice are given unilateral, numbers are rounded to the next 1000. Numbers in mice are means of 10 animals. CE, coefficient of error; *N*, number of cells measured; NA, not assessed.*

### Proliferative Potential and Cell Division is Largely Restricted to Early Stages

The number of cells expressing MCM2 were slightly higher than those of Ki67+ proliferating cells. In neonates, 37% (SD 1.6) of all MCM2+ cells were actively proliferating (MCM2+/Ki67+ cells), whereas in adults, 66% (SD 16.7) of the MCM2+ expressed Ki67 (**Table 2**; **Figure 2A**). The proportion of dormant cells with replication potential (MCM2+/Ki67-) cells were therefore nearly twice the size in neonates than in adults. Except for a few cells in neonates, Ki67+ cells that were negative for MCM2 were not detected. In neonates, 30% (SD 10.5) of all proliferating cells can be assigned to the IPC stage (Ki67+/Tbr2+, **Table 2**; **Figure 2B**). Interestingly, this value was maintained from birth to late adulthood [*b* = -0.28, *t*(10) = -0.87, *p* = 0.408]. Proliferation at the stage of DCX+ young neurons was almost absent as DCX+ cells co-expressing Ki67 were extremely rare (**Figure 2C**). At this stage, also proliferative potential waned as only 6% (SD 3.9) of all MCM2+ cells co-localized with DCX (**Table 2**). Absolute numbers of double-positive DCX+/MCM2+ cells were low and corresponded to only 2% of all DCX+ cells. Altogether, our results indicate that the majority (∼70%) of actively dividing, Ki67+ cells proliferate earlier than the Tbr2+ IPC stage in adult marmosets. Further characterization of the proliferating cell population using Ascl1 (Mash1) antibodies in this primate species was attempted but not successful in our hands as also reported by others (Bunk et al., 2011).

### Similar Numbers of CR+ and DCX+ Cells with Very Limited Overlap

In adults, numerous CR+ cells in the subgranular layer could be observed, and their numbers were in the range of those of DCX+ young neurons (see **Table 2**). The numbers of CR+ immature granule cells declined steadily with age and at a similar rate as DCX (see above). The numbers of DCX+ young neurons co-expressing CR was very low (estimated to <200 cells at the most, see **Table 2**; **Figure 2D**) and on average accounted for less than 0.4% (SD 1.5) of all DCX+ cells. Measurements of soma sizes of CR+ and DCX+ cells showed that CR+/DCX- cells had an average volume of 923 μm^3^ (*N* = 559, SD 629), CR-/DCX+ have an average volume of 435 μm^3^ (*N* = 208, SD 198). Sample size for CR+/DCX+ cells (*N* = 5) was too small to derive reliable estimates using the Nucleator.

### Granule Cells Heterogeneity along the Septo-Temporal Axis in Marmosets

In marmosets, a differential distribution of the two morphologically distinct granule cell types (**Figure 3A**) was observed. Large-sized granule cells with one distinct and large nucleolus and large cytoplasm were more uniformly distributed along the hippocampal longitudinal axis (**Figure 3B**) than small granule cells (**Figure 3C**). Small-sized granule cells with less distinct smaller or multiple smaller nucleoli and very narrow rims of cytoplasm exhibited a strong septo-temporally graded distribution. Interestingly, large granule cells in neonate marmosets were very similar to those in adults, both in terms of their septo-temporal distribution and their numbers (for statistics see above). In contrast, the number of small granule cells was markedly lower in neonates. The radial distribution of large granule cells was, however, very different between neonates and adults. Whereas the larger cells are mainly found in the outer half of the granule cell layer in neonates (**Figure 3A**’), they were found intermingled with small granule cells in the adults (**Figure 3A**”).

### Neurogenesis Level in Marmosets and Rodents

**FIGURE 4:**
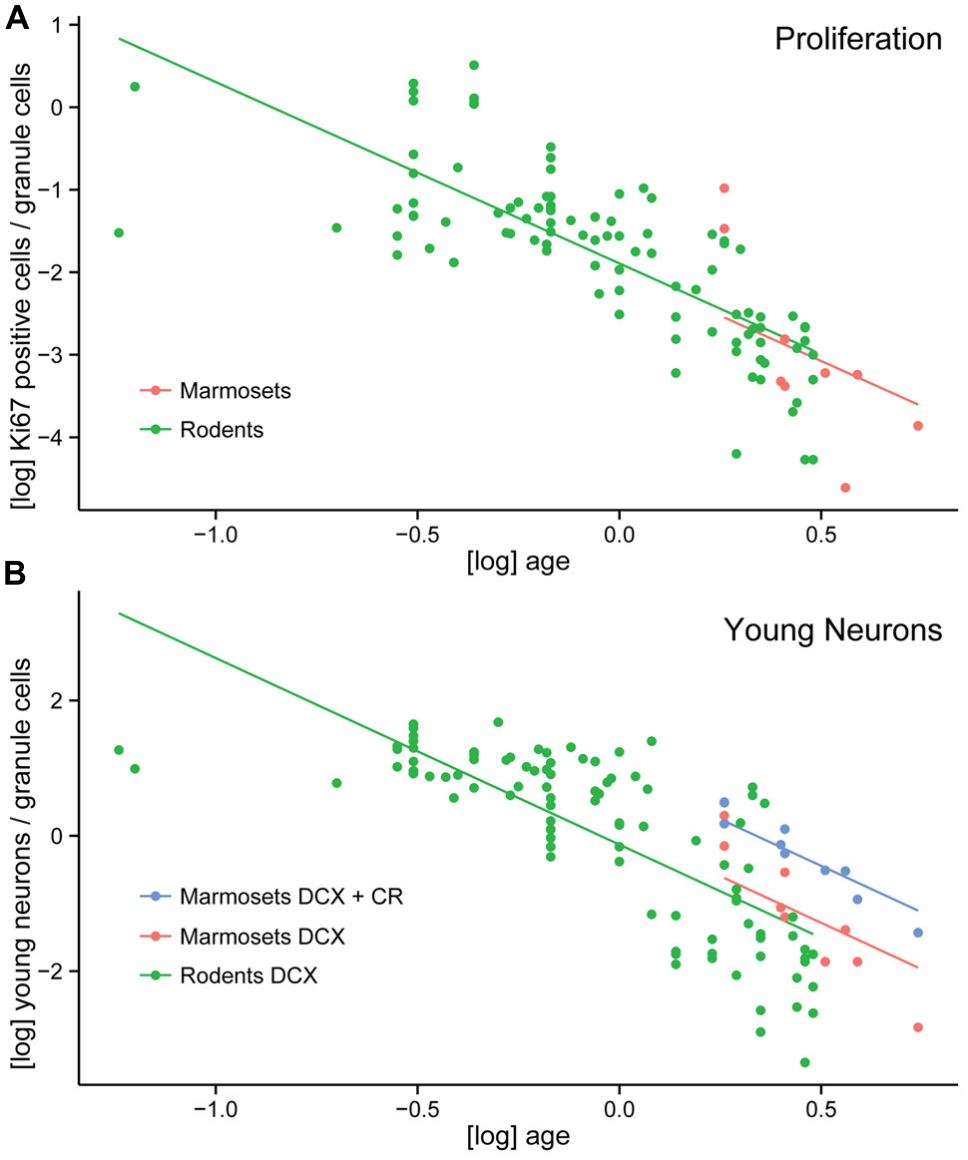
**Neurogenesis comparison between marmosets and rodents.(A)** Log-transformed number of proliferating cells per total granule cells (normalized proliferating cells) do not differ between a large number of rodents and marmosets. **(B)** Increased numbers of young neurons in marmosets compared to rodents are only apparent if the non-overlapping DCX+ and CR+ cell populations are pooled in the primate. Normalized numbers of DCX alone do not differ between rodents and marmosets.

In order to compare neurogenesis-related cell counts between species, we calculated the percentage of proliferating cells (Ki67+) and young cells of the neuronal lineage (DCX+ or PSA-NCAM+) relative to granule cells in the marmosets and a large set of rodent data (*N* = 98, Amrein et al., 2011, 2014; Cavegn et al., 2013). Neonate marmosets were excluded from the analysis due to leverage (Amrein et al., 2011). Corrected for age, the log-transformed normalized Ki67-value did not differ between marmosets and rodents [*F*(1,105) = 0.104, *p* = 0.75, **Figure 4A**]. Similarly, the normalized values of young neuronal cells positive for DCX or PSA-NCAM were not significantly different between marmosets and rodents [*F*(1,105) = 0.49, *p* = 0.49]. Only after pooling the non-overlapping stages of young neurons characterized by the expression of either DCX+ or CR+ cells in marmosets, marmosets showed a significantly higher value for young, immature neurons than rodents [*F*(1,105) = 11.6, *p* = 0.001, **Figure 4B**].

## Discussion

**FIGURE 5:**
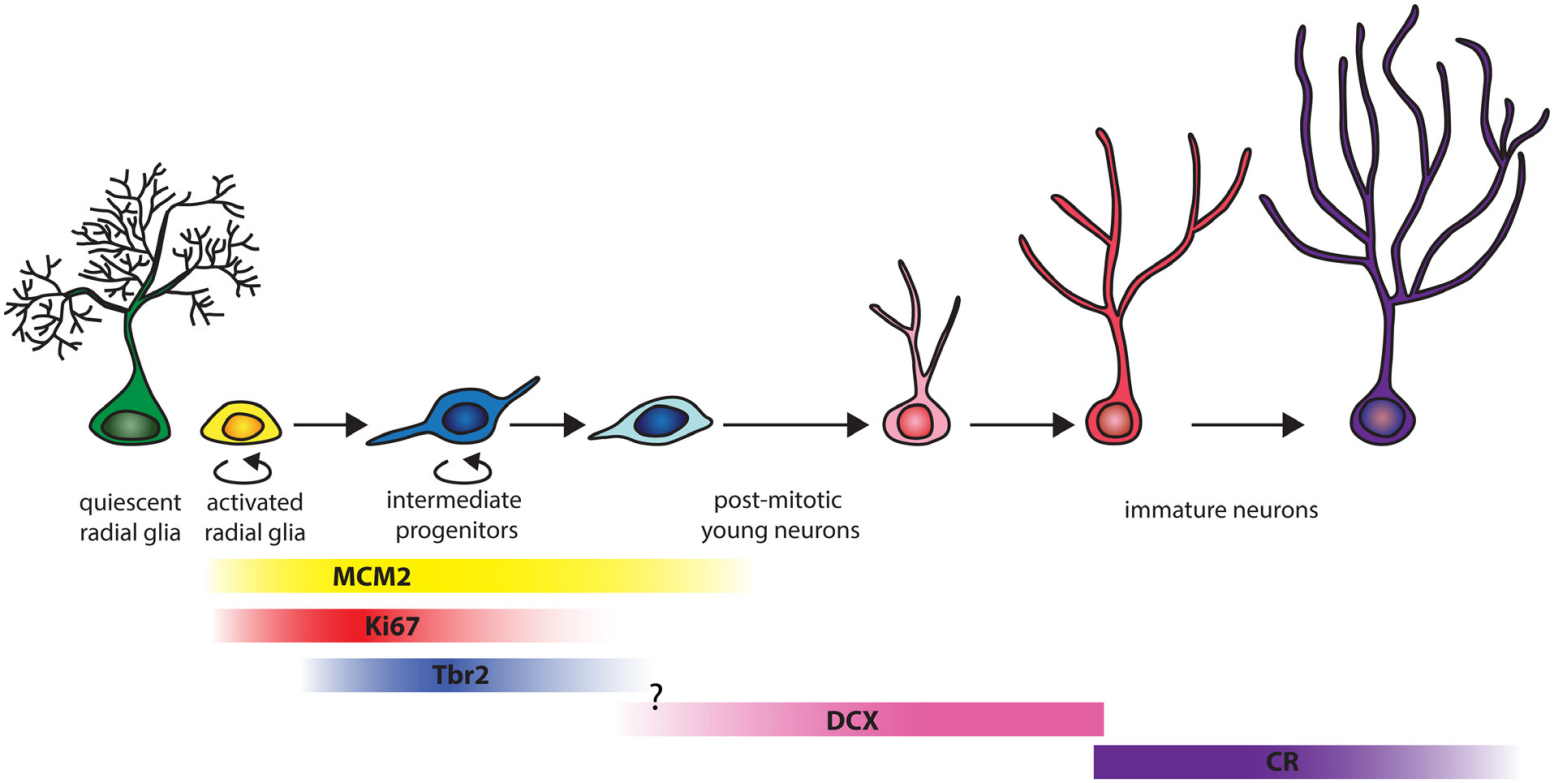
**Schema of the progression of hippocampal neurogenesis in adult marmosets.** The figure, adapted from Song et al. (2013), takes into account only markers used in the present study. In adult marmosets, around 66% of the replication competent MCM2+ cell co-label with Ki67. MCM2 expression extends until later stages, as 6% of the MCM2+ cells co-express DCX. Of all proliferating Ki67+ cells, around 30% can be assigned to the early intermediate precursor stage when cells express Tbr2. Proliferation at the stage of DCX+ young neurons is almost absent. The transition between Tbr2 and DCX was not examined in this study. The population size of DCX+ and CR+ cells are on average four times larger than those of MCM2, Ki67 or Tbr2, with minimal (0.4%) overlap between DCX+ and CR+ stages. CR+ cells therefore represent a distinct, prominent young neuron population.

The quantitative data presented here show similar septo-temporal distributions of granule cells, proliferating cells, and young neurons in the hippocampal dentate gyrus of marmosets and mice. The progression of neurogenesis in the adult marmosets differs compared to rodents with respect to the stage-specific proliferation activity and the protracted differentiation in the later stages of neuronal maturation as summarized in **Figure 5**.

### Dentate Gyrus Long-Axis Comparison between Marmoset and Mouse

Some morphological differences between humans and rodents along the septo-temporal axis of the hippocampus have recently been summarized by Strange et al. (2014). These differences raise questions pertaining to possible partitions within the hippocampus of the marmoset, a small simian primate with a largely lissencephalic brain. While more granule cells are found in the septal than in the temporal region in the rodent hippocampus (Gaarskjaer, 1978; Bekiari et al., 2015), granule cell numbers in the human hippocampus are highest anterior (temporal in rodents) and decline toward its posterior end (septal in rodents; Boldrini et al., 2013). Volumetric measurements of the baboon hippocampus indicate a similar septal to temporal gradient (Wu et al., 2014). Considering the gross anatomy of the primate hippocampus, the difference between rodents and primates is not surprising. The farthest anterior segment of the human and primate (Old World primates such as baboon, rhesus, long-tailed, and vervet monkey) hippocampus folds backward dorsally and forms the uncus. Coronal sections in the anterior region of their hippocampi can thus contain dentate gyrus layers before and after the curvature as illustrated in Seress et al. (2008), resulting in high anterior/temporal granule cell numbers. Folding of the temporal hippocampus is not seen in rodents and is not present in marmosets. The long axis of the marmoset hippocampus is fairly straight, starting ventromedial and running in a smooth, laterally bending arch toward the retrosplenial cortex [see also the web-based digital atlas of the marmoset brain presented by Tokuno et al. (2009)]. A folding in the uncus as in humans and Old World primates is not seen, while the mid-body and tail of the marmoset hippocampus are positioned similar to other primates. Consequently, the appearance of the granule cell layer in the marmoset dentate gyrus is a conglomerate of primate and rodent features and actually very similar to the straightened mouse hippocampus presented here. Quantitatively, granule cell numbers are lower in the temporal third than in the septal third in both marmosets and mice. This gradient is more pronounced in marmosets than in mice. These similarities further extend to the cell numbers that reflect adult hippocampal neurogenesis. Hippocampal neurogenesis, confirmed early in marmosets (Gould et al., 1998), exhibits a septo-temporal gradient similar to that in mice. Marmosets and mice contain a higher absolute number of DCX+ young neurons in the septal region, while absolute numbers of proliferating cells are evenly distributed along the septo-temporal axis. Our results in mice are by and large similar to previous reports (Snyder et al., 2009; Jinno, 2011; Bekiari et al., 2015). Interestingly, marmosets and rodents have parallel septo-temporal gradient in both Ki67+ and DCX+ cells that are opposite to gradients seen in the hippocampus of carnivores. Foxes contain the highest absolute and relative neurogenesis in the temporal hippocampus (Huang et al., 2015), whereas marmosets and mice reveal absolute, but not relative highest neurogenesis in the septal hippocampus. The significance of the different functional weights of neurogenesis in the temporal (fox) or septal (rodents, marmosets) dentate gyrus has yet to be investigated.

### Proliferation Potential is Largely Preserved in the Aging Marmoset Dentate Gyrus

The presence of MCM2 in cells marks the pre-replicative stage in the G1 phase (summarized by Tye, 1999), whereas Ki67 protein is expressed during the active phases of cell cycle only (Gerdes et al., 1983; Starborg et al., 1996). Neonate marmosets differ clearly in the number MCM2+ cells from adults by a 100-fold difference, whereas the difference in the number of actually proliferating cells (Ki67+) is increased ‘only’ 33-fold, suggesting the presence of a large pool of “quiescent” but proliferation competent cells early after birth. Correspondingly, the percentage of MCM2+ cells that are cycling (MCM2+/Ki67+) is only twice as high in neonates as in adults. In adults, spanning the age from 20 months when marmosets have reached the age of first reproduction (Ross, 1991) until the oldest animal in our sample, which reached an age close to the average life span of male marmosets (Nishijima et al., 2012), the population of cells with replicative potential (MCM2+) or actually dividing cells (Ki67+) remains relatively stable. Similarly, a stable proliferation rate of neuronal progenitor cells was reported in aging cynomolgus monkeys (Aizawa et al., 2011). Considering the small sample size in our study, low p values for both Ki67+ and MCM2+ cells and negative slopes of the regression lines, it may be possible to identify an age-dependent decrease in a larger sample of adults. It is, however, safe to conclude that a decrease in the number of proliferating cells or cells with proliferation potential, if present, is less pronounced than that of cells of the neuronal lineage.

### DCX Defines Post-Mitotic Neurons in the Marmoset Hippocampus

Around 30% of the Ki67+ proliferating cells in adult marmosets can be assigned to the early intermediate precursor (IPC) stage, defined by expression of the transcription factor Tbr2 (Hodge et al., 2008). The percentage is close to that reported in a small sample of dentate Ki67+/Tbr2+ cells in marmosets (Azim et al., 2013), in which Tbr2+ cells are Pax6+ and therefore likely to be early progenitors of the neuronal lineage. Marmoset values are, however, markedly lower than the 93% proliferating Tbr2+ cells (Tbr2+/PCNA+) reported in mice (Hodge et al., 2008). Further quantification of the proliferating cells in marmosets shows that barely any of the DCX+ cells co-localize with Ki67. Thus, 70% of the proliferating, Ki67+ cells in the marmoset dentate gyrus appear to belong to cellular stages prior to Tbr2 expression. Data derived from the rhesus monkey indicate that proliferation at the early, activated radial glia stage (Type 1 cells by Taffe et al., 2010) is higher in primates than rodents. With the approach taken here, we cannot definitely exclude a proliferative stage between the Tbr2+ and DCX+ stage. However, in mice it has been shown that Tbr2 and DCX show extensive overlapping expression patterns (Hodge et al., 2008). Our findings in marmosets stand in contrast to the proliferation activity of immature DCX+ neurons in rats and mice, in which considerable percentages (between 20 and 70%) of proliferating cells co-label with DCX (Brown et al., 2003; Jessberger et al., 2005; Plümpe et al., 2006). While DCX+ cell very rarely proliferate in marmosets, they encompass a small population of cells (DCX+/MCM2+) that retains proliferation potential. Marmosets may also differ from the rhesus monkey, in which proliferation activity in the committed neuronal lineage has been reported (Taffe et al., 2010). In the later study, proliferating cells were analyzed using NeuroD1+/Ki67+/Sox2+ (Type-2b) and NeuroD1+/Ki67+/Sox2- (Type-3) co-labeling. NeuroD1 and DCX show extensive overlap in expression in mice (Steiner et al., 2006), which remains to be determined in future studies in marmosets. It also remains to be assessed if differences represent species-specific proliferation patterns, or if they are due to experimental challenges, as the animals in the Taffe study were subjected to daily behavioral testing. Altogether, our data emphasize for the first time that the DCX+ pool in marmosets are largely post-mitotic young neurons.

### Lineage Characterization and Prolonged Maturation at the Post-Mitotic DCX and CR Stage

Calretinin (CR) has been described as a transient marker for immature granule neurons in mice (Liu et al., 1996; Brandt et al., 2003). The proportion of DCX+ young neurons that co-label with CR varies from ∼27% in 2 month-old (Brandt et al., 2010) to ∼80% in 3 months-old C57BL/6 mice (Llorens-Martín et al., 2006). A co-expression of DCX and CR has been reported in humans as well (Knoth et al., 2010). In marmosets, the CR+/DCX+ cells are below one percent in young adults and disappear completely in adult and older animals. There are other mammals in which DCX+ cells do not co-express CR, e.g., in sengi (Slomianka et al., 2013), and lesser hedgehog tenrecs (Alpár et al., 2010). In bonnet monkeys (*Macaca radiate*), a reactive expression of CR in 10% of the BrdU+ young neurons has been described following electroconvulsive therapy (Perera et al., 2007). This corresponds to the increased CR+/DCX+ co-expression after experimentally induced epileptic seizures in mice (Domínguez et al., 2003). Although co-expression of CR with DCX in marmosets is minimal, both types of cells are equally affected by age-related changes, and CR expression as part of adult hippocampal neurogenesis has also been shown in experimentally naïve primates (Lavenex et al., 2009) including marmosets (Tauber et al., 2008).

The transient CR expression in neuronal maturation is important for the normal progression of neurogenesis, as the loss of CR in knockout mice leads to reduced proliferation and survival of young granule cells (Todkar et al., 2012). In contrast, increased CR expression in mice deficient for the alpha-isoform of calcium/calmodulin-dependent protein kinase II (alpha-CaMKII) leads to a dentate gyrus that remains in an immature stage. The endophenotype in these mice, named immature dentate gyrus (iDG), was linked to psychiatric disorders (Yamasaki et al., 2008). Several studies have identified the iDG as a conserved trait in the pathophysiology of schizophrenia and bipolar disorders (for review see Hagihara et al., 2013). Animal models in these fields are usually based on mice. Further validation in a primate model, such as marmosets, seems necessary because the sizes of the cell populations characterizing the iDG differ between rodents and primates due to the primate-specific prolonged maturation of young neurons as shown here. Our observations suggest that CR expression correspond to a further maturational stage during hippocampal neurogenesis in marmosets. Such an extended maturation time of newborn granule cells was previously reported in macaque monkeys by using the marker TUC-4 (Toad-64; Ngwenya et al., 2006), which has an expression window similar to CR. Accordingly, a prolonged maturation in marmosets becomes apparent if the sum of the non-overlapping DCX+ and CR+ cells is considered, while normalized estimates of proliferation (Amrein et al., 2011) and neuronal differentiation – based on DCX alone – are very similar in marmosets, Old World primates and rodents. Previously reported data indicate that the number of CR+ cells in mice amounts to only one-third of DCX+ cells (Jessberger et al., 2005). Together with the substantial co-expression of the two markers in mice, a CR+/DCX- population of young granule cells should be very small in rodents.

### Granule Cell Heterogeneity

In several species, a morphological heterogeneity in the granule cell population has been reported. Findings pertain to the size of the granule cells in rhesus monkey and baboons (Seress and Frotscher, 1990; Jabès et al., 2010), foxes (Amrein and Slomianka, 2010; Huang et al., 2015), and rats (Bayer, 1982). An earlier report found that 20% of granule cells belong to a larger subpopulation in adult rhesus monkey and baboons (Seress and Frotscher, 1990), which is close to the average of 15% observed in marmosets. Heterogeneity has also been reported for variations in apical and/or basal dendrites in mice, primates and humans (Seress and Mrzljak, 1987; Seress and Frotscher, 1990; Becker et al., 2012), and for protein expression in granule cell axon terminals in mice (Rekart and Routtenberg, 2010). The functional significance of morphological granule cell diversity remains unclear, but it is intriguing that even among the adult-born granule cells, two distinct groups of neurons can be differentiated based on their spike responses (Brunner et al., 2014). In the animals sampled here, the numbers of large granule cells did not change with age, while their distribution within the granule cell layer was age-dependent. Large granule cells are almost exclusively found in the external granule cell layer in neonates, e.g., close to the molecular layer. In adults, they are intermingled with smaller sized granule cells throughout the granule cell layer. Larger cells in neonates are also more prominent in the suprapyramidal than in the infrapyramidal blade. Age-related changes and preferred locations suggest that the distribution of large granule cells resembles the sequence of granule cell layer formation in rats (Altman and Bayer, 1990). Data presented in macaques (Jabès et al., 2010) can also be interpreted in terms of a successive generation of initial large cells, i.e., an early peak of cells that are larger than the average adult granule cells, followed by the generation of the smaller cell population.

In summary, we found that the anatomy of the marmoset dentate gyrus greatly facilitates direct comparison of adult hippocampal neurogenesis between primates and laboratory rodents. However, previously unknown primate-specific features in the adult generation of dentate granule cells were described. These included the dramatic restriction of proliferation activity to earlier stages of the lineage, and the temporal separation of DCX and CR expression.

## Conflict of Interest Statement

The authors declare that the research was conducted in the absence of any commercial or financial relationships that could be construed as a potential conflict of interest.
